# Microarray analysis on the lncRNA expression profile in male hepatocelluar carcinoma patients with chronic hepatitis B virus infection

**DOI:** 10.18632/oncotarget.12732

**Published:** 2016-10-18

**Authors:** Jianjun Niu, Yong Lin, Pingguo Liu, Yiwen Yu, Chenghao Su, Xiaomin Wang

**Affiliations:** ^1^ Zhongshan Hospital, Medical College of Xiamen University, Xiamen, China; ^2^ Fujian Provincial Key Laboratory of Chronic Liver Disease and Hepatocellular Carcinoma, Xiamen, China; ^3^ Xiamen Center for Disease Control and Prevention, Xiamen, China

**Keywords:** hepatocellular carcinoma, lncRNA, HBV infection, male, expression profile

## Abstract

Long non-coding RNAs are involved with development and progression of cancer, and the advance of microarray technology allows the researchers to investigate the complete expression profile of lncRNA in various kinds of sample. We enrolled 5 male primary HCC cases with chronic HBV infection and the HCC and normal tissues have been obtained during the resection surgery. After total RNA extraction, the lncRNA microarray analysis was conducted to determine the lncRNA and mRNA expression signals. 612 lncRNAs and 1,064 mRNAs were significantly up-regulated in HCC tissue while 656 lncRNAs and 1,532 mRNAs were down-regulated in HCC tissues. Compared with normal tissues, *XLOC_007433* (fold change: 12.80) and *AC144449.1* (fold change: 27.20) were the most over- and under-expressed lncRNAs in HCC tissues. As for the mRNA, *THBS4* (fold change:41.13) and *CXCL14* (fold change: 58.03) were the most over- and under-expressed mRNAs in HCC tissues when comparing with their normal counterparts. In total, 4,552 pairs of lncRNA-mRNA were identified and the co-expression network was constructed. Moreover, the gene ontology enrichment analysis showed that the significantly different transcript between HCC and normal tissues were mainly associated with response to wounding, inflammatory response, protein hetrodimerization activity, response to stress which involved with biological process and molecular function. The pathway analysis suggested that the most significant pathways consisted of alcoholism, regulatory RNA pathways and RNA polymerase transcription. Several novel differentially expressed lncRNAs and mRNAs were identified in the present study.

## INTRODUCTION

Hepatocellular carcinoma (HCC) is the one of the most fatal among all kinds of cancer, roughly the fatality reached 0.95. The early detection and surgical resection would help to improve the outcome with a 5 year overall survival of 39% approximately [1]. However, most of HCC cases are not discovered and treated until the advanced stage or severe symptoms occurred. Due to the degenerated liver function, distant metastasis, and possible cirrhosis, liver resection is only available for less than 30% of HCC cases [2]. Even if some local therapies of HCC were introduced, including ethanol ablation, radiation therapy, and transcatheter aterial chemoembolization, the 5 year overall survival for HCC cases with any stage is only about 15% [3]. Therefore, it is of great importance to identify the risk factors of HCC and provide scientific evidence for cancer prevention. With decades of efforts, chronic hepatitis B virus (HBV) infection [4], hepatitis C virus infection [5], excessive consumption of alcohol [6], and aflatoxin exposure [7] were generally acknowledged as the risk factors of HCC. Particular in China, the HBV infection rate is about 7.18% in general population [8], and the high incidence of HCC in China can be attributed to the high infection rate. A large number of chronic HBV patients in China would continue to progress and possibly developed to cirrhosis and even HCC. The high incidence, high fatality and poor outcome combined together placed heave burden to both the patients' family and healthcare system of China.

Currently, the onset of HCC is thought to be a complicated process involved with multiple factors, including environmental factors, viral infection and genetic susceptibility. Furthermore, the statistics indicated that approximately 3%-8% of chronic HBV cases would progress to HCC eventually [9], which suggested that the mutations on HBV genome [10] and the genetic variation on human genome would alter the HCC risk together. Recently, researchers have shown an increased interest in investigating the association between long non-coding RNA (lncRNA) profile and the onset of cancer [11]. Advances in RNA sequencing technologies have discovered the existence of non-coding RNAs which comprised the majority of the transcriptome. By definition, lncRNA refers to the large and diverse class of transcribed RNA with a length of more than 200 nucleotides that lacks protein-coding potential [12]. LncRNA has been proved to possess multiple properties, including regulation of gene transcription, chromatin modification, and epigenetic regulation [13]. Growing evidence demonstrated that thousands of lncRNAs with aberrant expression are associated with different kinds of cancer, including HCC [14]. Previous study conducted among three male HCC cases has revealed that 8 lncRNAs were differentially expressed when comparing the HCC tissues with adjacent normal counterparts [15]. Such approach with remarkable findings, however, sample size was relatively small and HBsAg status among the enrolled subjects were not limited, and has failed to address the different expression profile properly. It is generally acknowledged that the HBV-induced HCC involves the integration of HBV fragment and hepatocellular genome, immune response caused chronic and persistent inflammation, which would definitely express different profile when comparing with non-HBV induced HCC. Xiamen ranks the third position in HCC prevalence with 49.57/100,000 [16], according to the cancer statistics released by China National Cancer Center. High chronic HBV infection rate in general population which was 13.79% leads to the high prevalence of HCC [17]. Moreover, comparing with national average level above mentioned, the HBV prevalence in Xiamen population was almost one fold higher. In this study, we attempted to investigate the expression profile on lncRNA among 5 male HBV induced HCC cases by using microarray analysis, thus we can provide scientific evidence for the male individuals with chronic HBV infection which have been identified as the high risk population of HCC.

## RESULTS

### Baseline demographic and clinical characteristics of 5 HCC cases

5 male HCC cases with chronic HBV infection undergone liver resection have been enrolled in our study between November 2015 to January 2016. The tissue samples have been acquired during operation in accordance with the procedures stated in materials and methods section. The demographic and clinical characteristics of enrolled 5 subjects were demonstrated in Table 1. As can be seen, the average age was 42.8 years, and the average duration of HBV infection was 12.6 years. The average tumor size was 5.86 cm, and 3 subjects have AFP level higher than 400 ng/ml. As for the liver function parameters, 3 subjects showed elevated AST level, and 4 have higher AST level when comparing with reference range, but only 1 subject showed elevated total bilirubin level.

**Table 1:** Baseline demographic and clinical characteristics

Subject ID	1	2	3	4	5	Average
Age (Year)	39	34	37	36	68	42.8
Duration of HBV infection(Year)	15	12	15	11	10	12.6
Tumor Size(cm)	6.3	5	10	3	5	5.86
AFP (ng/ml)	125718	4.74	5652	4.33	10979	28471.61
ALT(U/L)	30.4	91	33	53.5	43	50.18
AST(U/L)	45.9	53	99	26.2	60	56.82
Total bilirubin(μmol/L)	14.3	7.5	4.6	13.8	17.2	11.48

### Overview of the lncRNA and mRNA expression profile in HCC and normal tissues

**Figure 1:**
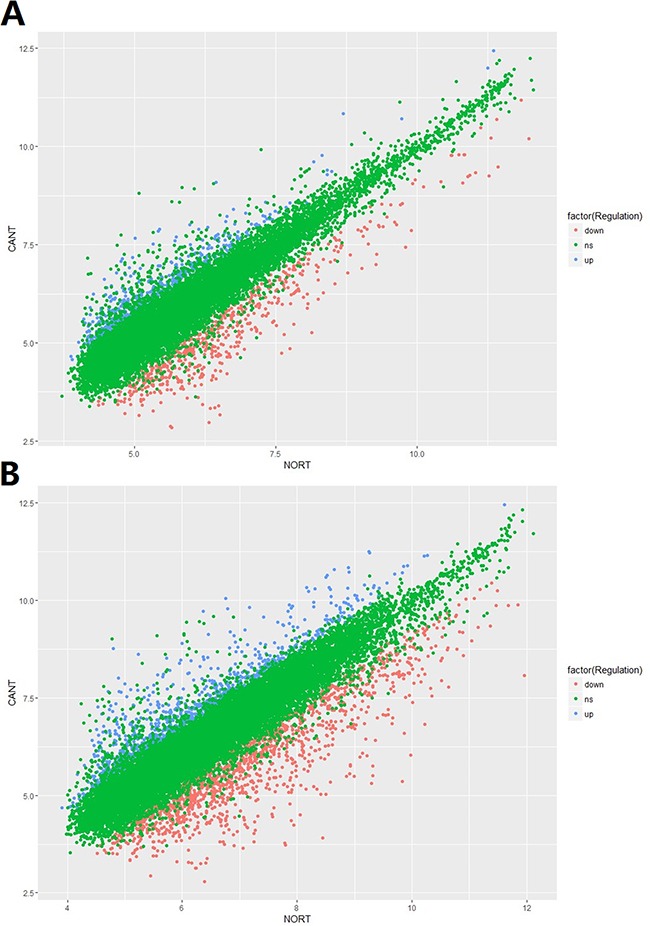
**The scatter plot of A.** lncRNA and **B.** mRNA expression signals in HCC and normal tissues.

**Figure 2:**
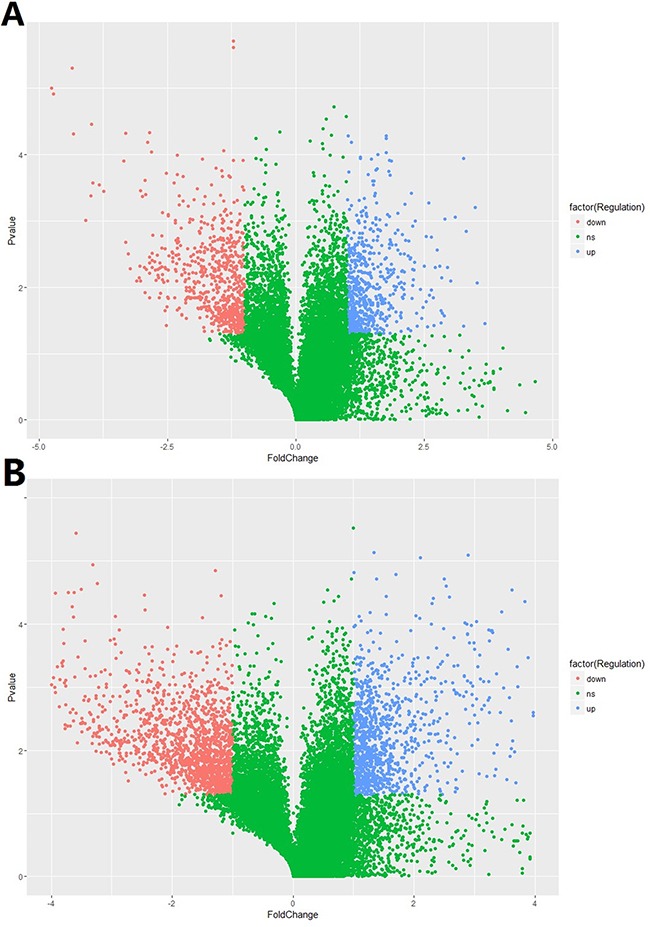
**The volcano plot of A.** lncRNA and **B.** mRNA expression signals in HCC and normal tissues.

In total, 15,328 lncRNAs and 21,717 mRNAs were found to be differentially expressed when comparing the HCC tissues and normal tissues in 5 enrolled subjects. Of these above mentioned RNAs, 612 lncRNAs and 1,064 mRNAs were significantly up-regulated (≥2 fold change and no less than 3 biological replicates) in HCC tissue while 656 lncRNAs and1,532 mRNAs were down-regulated in HCC tissues. Compared with normal tissues, *XLOC_007433* (fold change: 12.80) and *AC144449.1* (fold change: 27.20) were the most over- and under-expressed lncRNAs in HCC tissues. As for the mRNA, we found that *THBS4* (fold change:41.13) and *CXCL14* (fold change: 58.03) were the most over- and under-expressed mRNAs in HCC tissues when comparing with their normal counterparts. The scatter plot of differentially expressed lncRNAs and mRNAs was demonstrated in Figure 1 and the volcano plot was showed in Figure 2.

**Figure 3:**
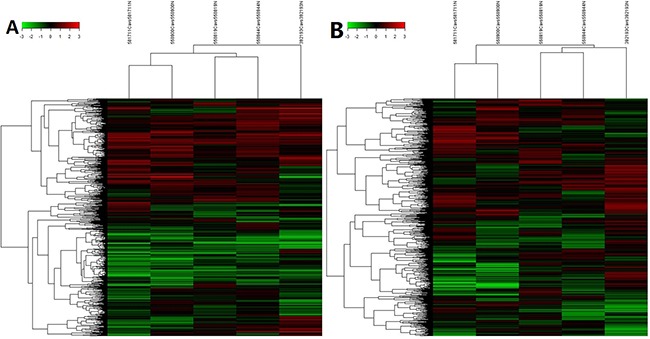
**Heat maps of differential expression and hierarchical clustering of A.** lncRNA and **B.** mRNA in HCC and normal tissues.

Based on the expression level of all tested RNAs in microarray analysis, we performed a hierarchical clustering analysis to group lncRNAs and mRNAs, allowing us to hypothesize the relationship among samples. The denodrogram in Figure 3 demonstrated the relationships of the lncRNA expression profiles between HCC tissues and normal tissue (Figure 3A) and mRNA expression profiles were showed in Figure 3B.

### Classification of differentially expressed LncRNAs

**Figure 4:**
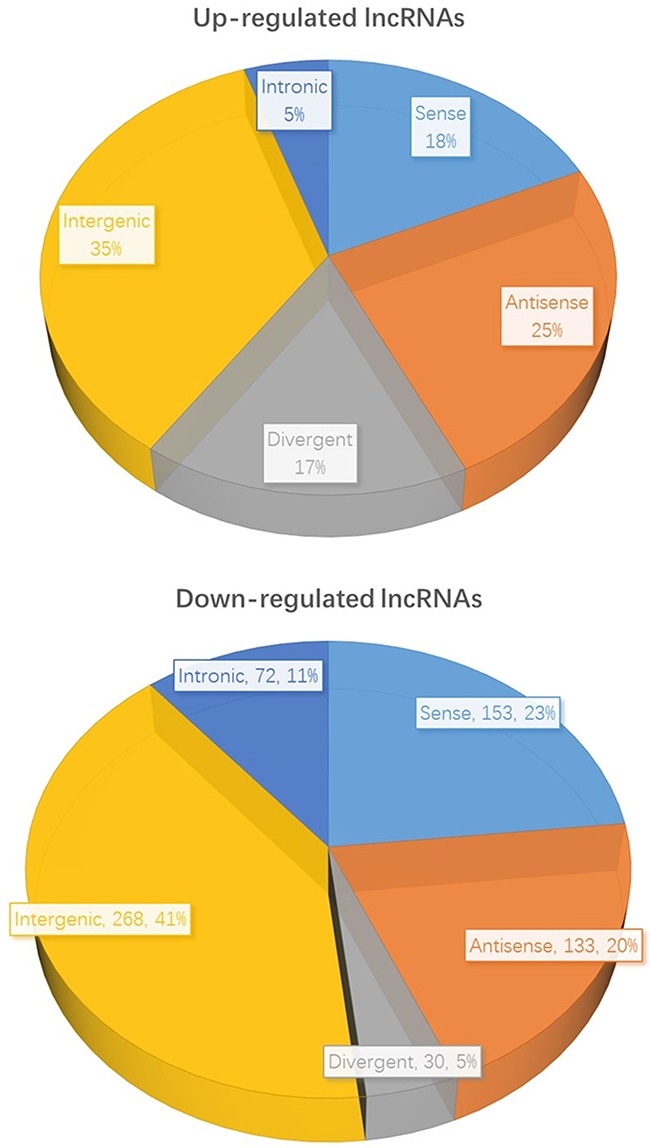
The classification of differentially expressed lncRNAs in HCC and normal tissues

We further classified the 612 over-expressed lncRNAs and 656 down-regulated lncRNAs in accordance with their different features, including genome location and context, exerted effect on DNA, and functioning and targeting mechanisms. The detailed results were showed in Table 2 and Figure 4. As can be seen, we found that 112 up-regulated and 153 down-regulated sense lncRNAs in the comparison of expression profile between cancerous and normal tissues. As for antisense lncRNAs, 150 were up-regulated and 133 were down-regulated. The profiling data also suggested 103 divergent lncRNAs were up-regulated while 30 were down-regulated. 215 intergenic lncRNAs were over expressed and 268 were under expressed. Furthermore, we identified 32 up-regulated intronic lncRNAs and 72 down-regulated, respectively.

**Table 2:** Classification of differentially expressed lncRNAs in comparing HCC tissues and normal tissue

lncRNA Classification	Up-regulated	Down-regulated	Total
Sense lncRNA	112	153	265
Antisense lncRNA	150	133	283
Divergent lncRNA	103	30	133
Intergenic lncRNA	215	268	483
Intronic lncRNA	32	72	104
Total	612	656	1268

### Construction of lncRNA-mRNA co-expression network

**Figure 5:**
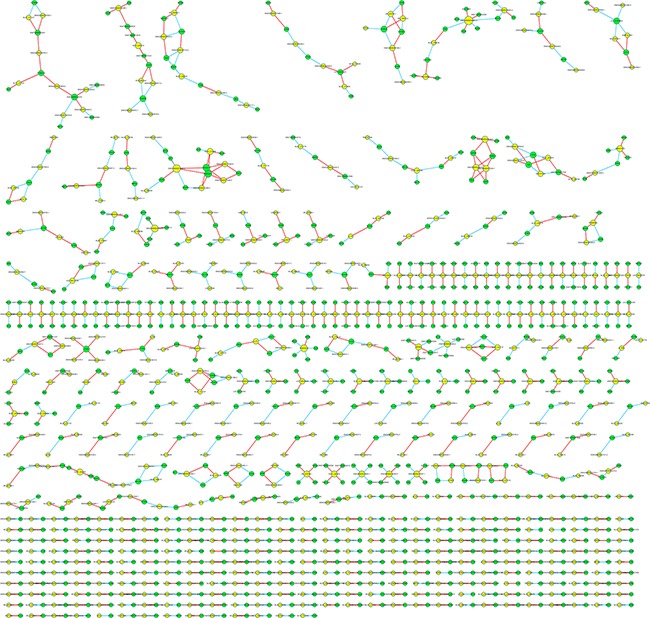
The lncRNA-mRNA co-expression network in Top 1000 correlated pairs

In order to investigate the correlation between differentially expressed lncRNA and mRNA, the lncRNA-mRNA co-expression network was constructed based on the correlation analysis and only those gene pairs with an absolute value of Pearson correlation coefficients not less than 0.99 were included. In total, 4,552 pairs of lncRNA-mRNA were identified in accordance with above mentioned criteria. We further selected the top 1000 among all significant correlations to draw the co-expression network (See Figure 5). The network suggested that one mRNA can be correlated with several lncRNAs and vice versa, however, we did not observe significant cluster by visual inspection. In particular, the most over-express lncRNA *XLOC_007433* were positively correlated with the expression of *HLA-DQB1, CFD, MSR1, LPAR5, GRAP2* and *MBNL2,* while inversely associated with *MAP3K13* and *MBNL2*. The correlation analysis found no mRNA was correlated with the most under-expressed lncRNA *AC144449.1.* The most up-regulated coding gene *THBS4* was correlated with 2 lncRNAs, namely *uc.77-* and *ENSG00000249042.1.* As for the *CXCL14* which has been identified as the most down-regulated gene, only *ENSG00000232593.2* was inversely correlated with the expression of it.

### Gene ontology (GO) and pathway analysis results

**Figure 6:**
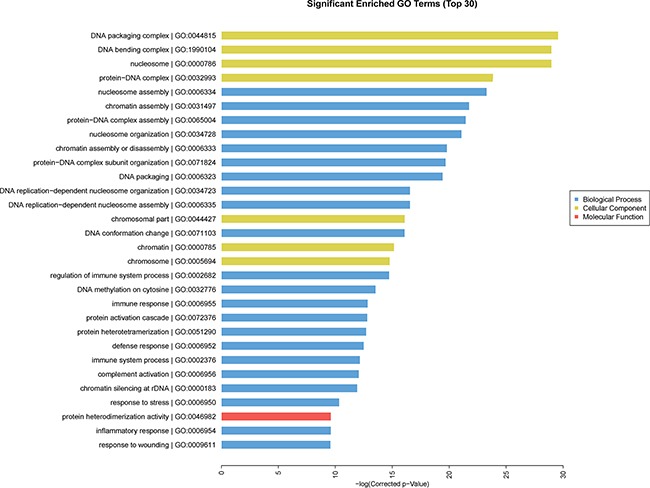
GO enrichment analysis of differentially expressed of mRNA

**Figure 7:**
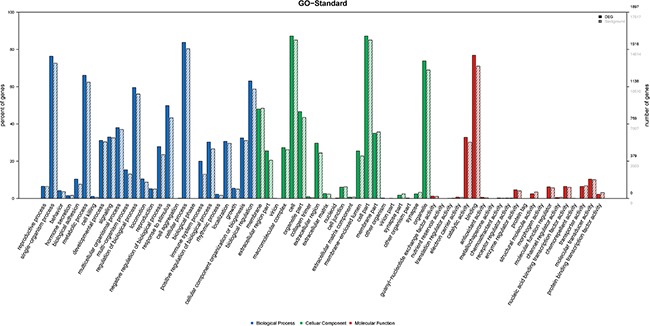
GO analysis of level 2 function of differentially expressed of mRNA

**Figure 8:**
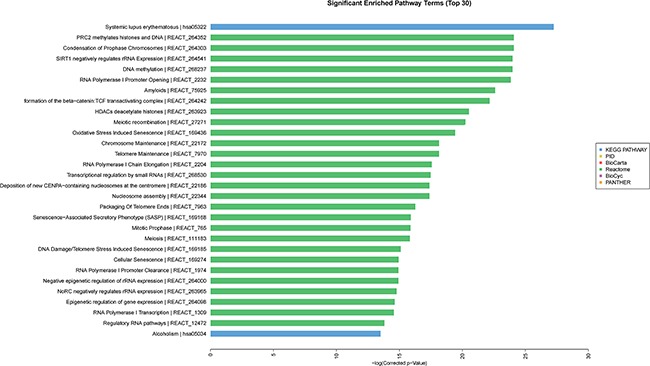
Pathway analysis of differentially expressed of mRNA

GO analysis was conducted among all differentially expressed mRNAs to identify the function of coding transcripts. Through the analysis we revealed that the significantly different transcript between HCC and normal tissues were mainly associated response to wounding (GO:0009611), inflammatory response (GO:0006954), protein hetrodimerization activity (GO:0046982), response to stress (GO:0006950) which involved with biological process and molecular function, and the detailed results were presented in Figure 6. Moreover, we found that cellular process (GO: biological process), localization (GO: biological process), extracellular matrix (GO: cellular component), and extracellular region complex (GO: cellular component) were the most enriched terms (See Figure 7). The pathway analysis suggested that the most significant pathways consisted of alcoholism (hsa05034), regulatory RNA pathways (REACT_12472) and RNA polymerase transcription (REACT_1309) and the detailed result of pathway analysis was showed in Figure 8.

## DISCUSSION

As reported by the previous studies, the incidence of HCC in male population was about 3 times higher when comparing with female counterparts in global range [18]. The gender difference in HCC incidence could partly attribute to the higher prevalence of excessive alcohol consumption, cigarette smoking in men. But more importantly, male population is prone to infect with HBV or HCV by sexual transmission or blood transmission, therefore, combined these evidences together, male individuals aged 45-65 have been identified as the high risk population of HCC. Given the high prevalence of chronic HBV infection in China, especially in the male population without the protection of universal HBV vaccination program, it is of great importance to investigate the underlying genetic mechanism of HCC among this high risk population. To achieve that, we enrolled 5 male HCC cases with chronic HBV infection and performed the lncRNA microarray analysis in cancerous and normal tissue and bioinformatics were conducted to analyze the data.

Chronic HBV infection would possibly lead to the onset of HCC, and the underlying mechanism of it is different from those caused by other carcinogens, for instance, aflatoxin and alcohol. The major feature of HBV infection is the integration into host genome, and the integration can both be found in HCC tissue [19] and non-tumor tissue [20] from chronic HBV-infected patients. The random integration event has been acknowledged as the risk factor of developing HCC, because it is capable of disrupting cellular gene expression which is important for cellular growth and differentiation. A study revealed that the gene disruption, viral promoter-driven human transcription, DNA fusion and copy number alteration near the integration sites [21], indicating that the gene expression can be altered by HBV infection and consequently elevating the HCC risk. In our present study, we identified that 1,676 lncRNAs and 2,596 mRNAs were differentially expressed when comparing the HCC tissues with the normal counterparts. Unlike gene coding RNAs, lncRNAs are generally expressed in very low level, and the expression prolife of lncRNA in cancer development is specific [22]. Considering the large number of differentially expressed lncRNAs and mRNAs we identified in our samples, we can assume that the HBV-induced carcinogenesis is involved with the altered expression of various kinds of lncRNA and mRNA.

Surprisingly, both the most over and under expressed lncRNAs have not yet been associated with any cancer in reviewing previous researches, however, we did observe the positive correlation between *XLOC_007433* which is the most over-expressed in HCC tissues and *HLA-DQB1.*It is generally acknowledged that human leukocyte antigens (HLA) involved with defending viral infection and carcinogenesis. Our study examined the mRNA expression signal and found that the *HLA-DQB1* was significantly lower in HCC tissue. Although the *HLA-DQB1* has been found to be associated with some cancers, however, the precise role of it has not yet been identified, a recent meta-analysis suggested that the variations occurred in *HLA-DQB1* were related with the risk of HCC after pooling all available data [23]. These findings could partly support our assumption that among the chronic HBV carriers with compromised immunity, the chance of developing HCC is higher.

As for the gene coding RNAs, one unanticipated finding was that *THBS4* is the most up-regulated gene in HCC tissues. Generally speaking, *THBS4* belongs to the thrombospondin protein family which involved in diverse biologic processes given their potential to bind numerous proteins and serve as interaction platforms in the extracellular matrix [24]. The elevation of *THBS4* expression in HCC tissue was firstly reported according to our data, however, its expression has been associated with the invasion of breast cancer previously, and the evidence suggested that elevated *THBS4* expression contributes to the activated stromal response exhibited during tumor progression and this may facilitate invasion of tumor cells [25]. Although no previous publication supports the role of *THBS4* in HCC development, it is worth to investigate the underlying mechanism between *THBS4* and HCC, for the expression of *THBS4*can be up-regulated in tissue injury, remodeling, immune response and inflammation. *CXCL14* is a novel chemokine, and mainly stimulating cell migration that involved with immune surveillance, inflammation and cancer [26]. Our finding is consistent with the results of an animal experiment which suggested that *CXCL14* was significantly suppressed in HCC tissues of mice in vivo, moreover, *CXCL14* has been proved to be a tumor suppressor and capable of inducing tumor cell apoptosis through both the mitochondrial and nuclear apoptosis pathway [27]. A case-control study involved with 361 HBV-related HCC cases and 407 healthy controls also supports our finding, it has been revealed that the polymorphism in *CXCL14* was associated with the HCC progression, suggesting that *CXCL14* might alter the disease development by inhibiting tumor growth [28]. Hence it could conceivably be hypothesized that *CXCL14* could be an important tumor suppressor in HBV-related HCC, and investigation on the underlying mechanism should be conducted.

GO and pathway analysis showed that differentially expressed mRNAs are mainly involved with wounding, inflammatory response, protein hetrodimerization activity and response to stress. The results may be explained by the fact that viral protein generates inflammatory environment within the liver, and reactive oxygen species resulting oxidative stress causes widespread hepatic cell damage [29]. This also accords with our earlier observations, which showed that the alteration of global Th1/Th2-like cytokine expression was observed in metastatic HCC tissues when comparing with non- metastatic tissues, suggesting that inflammatory response is capable of promoting HCC metastasis [30].

Overall, we investigated the lncRNA and mRNA expression profile which are related to the onset of HCC in male chronic HBV subjects, and several novel differentially expressed lncRNAs and mRNAs were identified by using microarray analysis. Further research is necessary to reveal the molecular mechanism and biological function of lcnRNAs in HBV-related HCC.

## MATERIALS AND METHODS

### Sample collection and RNA extraction

This study was approved by the Ethical Committee of Zhongshan Hospital, Xiamen University. All subjects enrolled were physically signed the written consent before sample collection. Samples were acquired from 5 eligible HCC cases following these criteria: (1) pathologically diagnosed with primary HCC (ICD9-155); (2) male; (3) chronic HBV infection confirmed by ELISA prior to the onset of HCC; (4)permanent residents who lived in Xiamen over 10 years and aging from 20 to 79 years. Patients were excluded if any of the following conditions were met:(1) liver disease due to parasitosis, diabetes, fatty liver, metabolism disorders or severe cardiovascular diseases; (2) presence of cancers other than HCC; (3) autoimmune hepatitis or toxic hepatitis; (4) refuse to participate. Tissues acquired from 5 eligible HCC cases were divided into following two groups: primary HCC tissues (CA) and normal tissue distant from tumor edge for 5cm (NT). In total, 10 tissue samples were acquired during the liver resection and placed in liquid nitrogen pre-freezing RNase-free vial for 5 min, and stored at −78°C prior to RNA extraction. Tissue samples were subjected to RNA extraction using Trizol reagent (Invitrogen, MA, USA). The purity and concentration of RNA were measured from OD260/280 readings using a NanoDrop ND-1000, and the integrity was assessed using standard denaturing agarose gel electrophoresis. Only RNA extracts with total volume higher than 8μg undergone further analysis.

### Microarray analysis

The paired cancer and normal samples used to synthesize double stranded complementary DNA (cDNA), and the cDNA product was labeled and hybridized to lncRNA + mRNA Human Gene Expression Microarray V4.0 (CaptialBio Corp, Beijing, China) in accordance with the manufacturer's instructions. The microarray we used contains 40,916 human lncRNAs probes and 34,235 mRNA probes, and 4,974 Agilent control probes. Each RNA was detected by corresponding probes repeated for two times.

### Microarray imaging and data analysis

The data generated from lncRNA + mRNA microarray was analyzed for data summarization, normalization and quality control by using GeneSpring software version 12.0 (Agilent, CA, USA). In order to identify the differentially expressed genes, we employed threshold values of ≥2 fold change and a Benjamini-Hochberg corrected P value of ≤0.05. The data was log 2 transformed and median centered by genes using Adjust Data function of Multiexperiment Viewer software (Dana-Farber Cancer Institute, MA, USA). Further analysis, such as hierarchical clustering with average linkages was performed. Treeview software (Stanford University, CA, USA) composed by Java was employed to visualize the microarray results.

### Correlation analysis between lncRNA and mRNA

The network between lncRNA and mRNA was constructed based on the correlation analysis among differentially expressed lncRNA and mRNA. For each pair of genes, a Pearson correlation was estimated and the pairs with an absolute value of Pearson correlation coefficients not less than 0.99 were selected to draw the network by using Cytoscape. In network analysis, yellow node represents the lncRNA and green node represents the mRNA. Red lines indicate a positive correlation, and blue lines indicate an inverse correlation.

### GO and pathway analysis

GO analysis provides three structured networks of defined terms that describe gene product properties, including biological process, cellular component, and molecular function. Differentially expressed mRNAs between HCC tissue and normal tissue were included in GO term enrichment and pathway analysis based on the latest KEGG database. This analysis enabled us to identify the biological pathways for differentially expressed mRNAs in acquired samples.
